# Pyruvate dehydrogenase kinase 4‐mediated metabolic reprogramming is involved in rituximab resistance in diffuse large B‐cell lymphoma by affecting the expression of MS4A1/CD20

**DOI:** 10.1111/cas.15055

**Published:** 2021-07-28

**Authors:** Duanfeng Jiang, Qiuyu Mo, Xiaoying Sun, Xiaotao Wang, Min Dong, Guozhen Zhang, Fangping Chen, Qiangqiang Zhao

**Affiliations:** ^1^ Department of Hematology The Third Xiangya Hospital Central South University Changsha China; ^2^ Department of Hematology Affiliated Hospital of Guilin Medical University Guilin China; ^3^ Department of Hematology The Qinghai Provincial People’s Hospital Xining China; ^4^ Department of Hematology The Second Affiliated Hospital of Hainan Medical University Haikou China; ^5^ Department of Blood Transfusion The Third Xiangya Hospital Central South University Changsha China

**Keywords:** diffuse large B‐cell lymphoma, metabolic reprogramming, MS4A1/CD20, PDK4, rituximab

## Abstract

Diffuse large B cell lymphoma (DLBCL) heterogeneity promotes recurrence and anti‐CD20‐based therapeutic resistance. Previous studies have shown that downregulation of MS4A1/CD20 expression after chemoimmunotherapy with rituximab leads to rituximab resistance. However, the mechanisms of CD20 loss remain unknown. We identified that pyruvate dehydrogenase kinase 4 (PDK4) is markedly elevated in DLBCL cells derived from both patients and cell lines with R‐CHOP (rituximab plus cyclophosphamide, doxorubicin, vincristine, and prednisone) resistance. We found that overexpression of PDK4 in DLBCL cells resulted in cell proliferation and resistance to rituximab in vitro and in vivo. Furthermore, loss of PDK4 expression or treatment with the PDK4 inhibitor dichloroacetate was able to significantly increase rituximab‐induced cell apoptosis in DLBCL cells. Further studies suggested PDK4 mediates a metabolic shift, in that the main energy source was changed from oxidative phosphorylation to glycolysis, and the metabolic changes could play an important role in rituximab resistance. Importantly, by knocking down or overexpressing PDK4 in DLBCL cells, we showed that PDK4 has a negative regulation effect on MS4A1/CD20 expression. Collectively, this is the first study showing that targeting PDK4 has the potential to overcome rituximab resistance in DLBCL.

## Graphical abstract

High pyruvate dehydrogenase kinase 4 (PDK4) expression is associated with rituximab resistance in diffuse large B‐cell lymphoma (DLBCL) cells. Targeting PDK4 increases rituximab sensitivity against DLBCL cells. PDK4 has a negative regulatory effect on MS4A1/CD20 expression in DLBCL cells. PDK4 promotes proliferation and rituximab resistance in DLBCL cells by mediating metabolic reprogramming.

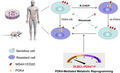

**Abbreviations**
ABC: activated B‐cell‐like
CCC: consensus cluster classification
COO: cell‐of‐origin
CT: computed tomography
DCA: dichloroacetate
DEG: differentially expressed gene
DLBCL: diffuse large B‐cell lymphoma
ECAR: extracellular acidification rate
EV: empty vector
GCB: germinal‐center B‐cell‐like
OCR: oxygen consumption rate
OE: overexpressing
OXPHOS: oxidative phosphorylation
PDK4: pyruvate dehydrogenase lipoamide kinase isozyme 4
qRT‐PCR: real‐time quantitative PCR
R‐CHOP: rituximab plus cyclophosphamide, doxorubicin, vincristine, and prednisone

## 1 INTRODUCTION

Diffuse large B‐cell lymphoma is the most common lymphoid malignancy, which comprises a heterogeneous group with pathophysiological, genetic, and clinical features.^1^ Most patients can be cured with R‐CHOP, which is the current standard regimen.^2^ However, approximately 40% of patients with DLBCL still experience therapeutic failure with R‐CHOP.^3^, ^4^, ^5^ This emphasizes the necessity to identify the molecular for providing new prognostic biomarkers and/or therapeutic targets.

The molecular heterogeneity of DLBCL is considered a major factor influencing the response to R‐CHOP therapy.^6^ The COO classification and the CCC capture largely different molecular aspects of DLBCL. The COO classification delineates subgroups of DLBCL into distinct transcriptional profiles: ABC, GCB, and unclassified.^7^ The CCC identifies DLBCL subsets including the B‐cell receptor/proliferation cluster, the OXPHOS cluster, and the host response tumors.^8^ Nutrient and energy metabolism in OXPHOS‐DLBCL are characterized by elevated oxidative phosphorylation and increased contribution of mitochondria to total cellular energy budget, while the “non‐OXPHOS” DLBCLs have greater glycolytic flux.^9^ However, whether distinct metabolic fingerprints influence DLBCL response to R‐CHOP remains unknown. Diffuse large B‐cell lymphomas, especially those previously treated with R‐CHOP Regimen, represent highly metabolically active tumors.^10^ Therefore, targeting DLBCL metabolic specificity might be a valuable therapeutic approach in the clinic, in particular for R‐CHOP low‐responder patients.

Rituximab, a chimeric mAb targeted against the pan‐B‐cell marker CD20,^11^ was reported to be affected by binding to CD20 and producing complement‐dependent cytotoxicity, Ab‐dependent cellular cytotoxicity, and apoptosis.^12^, ^13^, ^14^ However, loss of CD20 becomes a major obstacle for the retreatment of relapsed/refractory DLBCL with rituximab‐based regimens.^15^ MS4A1, which encodes CD20, rarely experiences nonsense and missense mutations in newly diagnosed samples, but these mutations increase significantly after rituximab‐associated therapy.^16^ In addition, acquirement of CD20 downregulation has been observed in patients after rituximab‐based therapies, which has been proven to be one of the most important reasons for rituximab resistance.^17^, ^18^, ^19^ Therefore, understanding the mechanisms of CD20 loss could contribute to the development of strategies for overcoming rituximab resistance in DLBCL.

Metabolic reprogramming plays an important role in tumor progression and drug resistance in multiple cancers.^10^, ^20^, ^21^, ^22^ Pyruvate dehydrogenase kinase 4 is a PDK isozyme that is highly expressed in cardiac and skeletal muscle, as well as being overexpressed in various tumors.^23^ Upregulation of the PDK family (PDK1‐4) is associated with aerobic glycolysis and chemoresistance through inhibition of the pyruvate dehydrogenase complex.^24^ Pyruvate dehydrogenase kinase 4 has been suggested as one of the most important factors controlling cell metabolism by directing carbon flux into glycolysis from OXPHOS.^25^, ^26^ Recently, DLBCL has been proved to be characterized by metabolic heterogeneity in different subtypes and different periods of therapy.^9^, ^10^ However, the roles of metabolic shift and its related mechanisms in rituximab resistance of DLBCL are not investigated.

The functional role of PDK4 in DLBCL has not been clarified. In this study, we identified that PDK4 was dramatically upregulated in rituximab‐resistant DLBCL cells. We further found that PDK4 has a strong correlation with MS4A1/CD20 expression. Further studies showed that PDK4 has a negative regulatory effect on MS4A1/CD20 expression. More evidence from in vitro and in vivo experiments confirmed that PDK4 plays a vital role in promoting DLBCL cell growth and rituximab resistance. In addition, PDK4‐mediated metabolic shift is involved in rituximab resistance. These results indicated that PDK4 can be a potential target for DLBCL therapy.

## 2 MATERIALS AND METHODS

### 2.1 Patients and tissue samples

The study was approved by the Ethics Committee of the Third Xiangya Hospital of Central South University. Informed consent was obtained from the patients according to the Declaration of Helsinki. Tissue samples and clinical data were obtained from patients who were diagnosed with DLBCL before treatment between December 2018 and August 2020. The diagnosis of DLBCL was confirmed by at least two pathologists in accordance to the WHO classification.^27^ The tissues were immediately frozen and stored in liquid nitrogen. Cryopreserved tissues contained both cancerous and paired distant normal tissues. This study was carried out in a retrospective series of 56 DLBCL cases with cryopreserved tissues, with follow‐up to December 2020.

### 2.2 Cell culture and reagents

The human DLBCL cell lines U2932, OCI‐ly7, and OCI‐ly8, and human normal B‐lymphocyte cell line GM12878 were obtained from the Cancer Research Institute of Central South University. The human DLBCL cell line SU‐DHL‐2 and the R‐CHOP‐resistant DLBCL cell line SU‐DHL‐2/R were obtained from Xiangya Hospital of Central South University and were characterized as reported previously.^28^ All cell lines were cultured in RPMI‐1640 (Gibco) with 12% FBS, 100 U/mL penicillin, and 100 mg/mL streptomycin.

Rituximab‐resistant DLBCL cell line OCI‐ly8/R was established as previously described.^29^ OCI‐ly8 cells were exposed to rituximab over a period of weeks to create rituximab‐resistant lines. Briefly, sensitive parental cell lines were cultured in RPMI‐1640 and once the log phase of growth was reached, cells were exposed for 24 hours to an increasing dose of rituximab (from 0.1 to 128 μg/mL). After 24 hours of incubation with rituximab, cells were centrifuged and recultured in fresh RPMI‐1640. Cells were then allowed to regrow for a minimum of 3 days, and once the log phase of growth was reached, the procedure was repeated for a total of 10 times. Rituximab was supplied by Roche Pharma and sodium dichloroacetate was supplied by Selleckchem.

### 2.3 Cell growth ability assay

For cell growth ability assay, 2000 cells in a 200‐µL volume were added to each well of a 96‐well plate and treated with rituximab (50 μg/mL) for indicated times. The Countess Automated Cell Counter (Thermo Fisher Scientific) was used to evaluate cell counts.The relative cell growth was the ratio of hour X to hour 0 using cell counts, eg, the relative cell growth of 0 hour was 1.

### 2.4 Cell viability assay

Cells were seeded in 96‐well plates (5 × 10^3^ cells per well) and exposed to different concentrations of drugs for 48 hours. Cell viability was assessed using CCK‐8 solution (Dojindo) by following the manufacturer’s instructions.

### 2.5 Cell apoptosis assay

Cells were stained by phycoerythrin‐conjugated annexin V and 7‐AAD (BD Biosciences) according to the manufacturer’s instructions. The stained cells were analyzed on a FACScan, and apoptotic cells were defined as annexin V‐positive cells.

### 2.6 Western blot analysis

Western blot analyses were carried out according to standard protocols. Anti‐PDK4, anti‐CD20, cleaved caspase‐3, and anti‐β‐actin were purchased from Affinity Biosciences.

### 2.7 Real‐time quantitative PCR

Total RNA was isolated from tissues and cells using the FastPure Cell/Tissue Total RNA Isolation Kit (Vazyme). Then 2 μg RNA was converted to cDNA with HiScript III RT SuperMix (Vazyme) according to the manufacturer’s instructions. Gene expression was analyzed using SYBR qPCR Master Mix (Vazyme) and LightCycler 480 (Roche) in a two‐step qRT‐PCR. The specific primers (Table S1) were synthesized by the Beijing Genomics Institute. Human B‐lymphocyte GM12878 cells were used as a calibrator. The mRNA relative levels of the target genes were calculated using the 2^−ΔΔCt^ method.

### 2.8 RNA sequencing

Total RNA was isolated from tissues as mentioned above. Total mRNA preparation and sequencing were carried out by the Beijing Genomics Institute.^25^, ^30^ Identification of DEGs was undertaken as previously described.^31^

### 2.9 Measurement of mitochondrial membrane potential

The mitochondrial membrane potential (Δψm) was determined using a JC‐1 staining dye assay kit (MedChemExpress) according to the manufacturer’s instructions. Briefly, cells were incubated with 1 μg/mL JC‐1 stain for 15 minutes in the dark at 37℃. Then cells were rinsed three times with PBS and subsequently observed and imaged using a fluorescence microscope (Olympus). The reduction of the red/green ratio is commonly used as an indication of apoptosis. The intensities of fluorescence were analyzed by ImageJ software.

### 2.10 Determination of glucose consumption, lactate production, and ATP levels

The glucose consumption, lactate production, and ATP level assays were carried out according to the previous study.^32^ Cells were cultured for 20 hours. The culture media were then harvested, and the lactate and glucose concentrations were measured using a lactate assay kit (BioVision) and glucose assay kit (Sigma‐Aldrich), respectively. The ATP levels were quantified using a colorimetric ATP assay kit (Beyotime) according to the instructions of the manufacturer.

### 2.11 Analysis of mitochondrial ATP synthesis and energy budget calculations

The rate of mitochondrial ATP synthesis was determined as previously described.^33^ Briefly, 2.5 × 10^5^ cells were resuspended in reaction mixture and incubated at 37℃ for 15 minutes. At 0, 5, 10, and 15 minutes, 50‐μL aliquots of the reaction mixture were quenched in 450 μL boiling buffer for 2 minutes. Then the quantity of ATP was determined using the colorimetric ATP assay kit. The rate of mitochondrial ATP synthesis was calculated from the difference in ATP content in the presence and absence of oligomycin. For energy budget calculations, the contribution of mitochondria and glycolysis to total cellular ATP was measured as previously reported.^9^, ^34^ For each cell line, nmol of ATP derived from glycolysis or OXPHOS were pooled and percent contributions to the total ATP production were calculated.

### 2.12 Measurement of ECAR and OCR

Cells were seeded on a 96‐well plate with a density of 5 × 10^4^ cells/well and treated as indicated. Cells were then treated with ECAR reagents according to the manufacturer’s recommendations (Abcam). The ECAR measurements were taken at 5‐minute intervals for a total assay time of 120 minutes by a microplate reader system (PerkinElmer) using excitation and emission wavelengths of 380 and 615 nm, respectively. The OCR was measured in real time using the XF24 extracellular flux analyzer instrument (Seahorse Bioscience) as described previously.^35^, ^36^ Protein concentrations were used to normalize the results.

### 2.13 Lentiviral infection

Two pairs of shRNA sequences (Tsingke Biotechnology) targeting human PDK4 were annealed and ligated into the Plk0.1‐puro lentiviral vector. The targeting sequences of PDK4 shRNA‐1 and shRNA‐2 were 5′‐ACTGCAACGTCTCT GAGGTG‐3′ and 5′‐AAGCAGATCGAGCGCTACTC‐3′, respectively. A scrambled shRNA was used as a control. Human PDK4 coding sequence (Vigene Bioscience) was cloned into the pcDNA3.1 plasmid to generate a pcDNA/PDK4 expression plasmid. The pcDNA3.1 was used as empty vector control for analysis. To generate PDK4 stable knockdown and overexpression cells, recombinant lentivirus was produced by transient transfection of HEK293T cells. The transfection efficacy was determined by qRT‐PCR and western blot analysis.

### 2.14 Immunofluorescence analysis

The cells were collected and placed on glass substrates and fixed with 4% paraformaldehyde for 20 minutes. The fixed cells were incubated with primary Abs overnight at 4ºC and stained with secondary Ab for 1 hour at room temperature in the dark, followed by counterstaining with DAPI (Sigma). The images were analyzed and captured by a confocal fluorescence microscope (Olympus).

### 2.15 Tumor xenografts in mice

All experiments involving animals were approved by the Institutional Animal Care and Use Committee of Central South University, China. Female B‐NDG mice (5‐7 weeks old) obtained from Jiansu Biocytogen Co., Ltd were used.^37^, ^38^, ^39^ The DLBCL xenograft mouse model was established through subcutaneous injection of 1 × 10^7^ PDK4‐overexpressing OCI‐ly8 cells (OCI‐ly8 PDK4 OE) or OCI‐ly8 cells transfected with empty vector (OCI‐ly8 EV) into the right flank of B‐NDG mice. At 10 days after the injection of the DLBCL cells, when the tumors became palpable, half of the mice derived from both the PDK4 OE group and EV group were treated with rituximab (12.5 mg/kg, equivalent to 225‐275 μg/mouse) injected intraperitoneally daily for 2 weeks; PBS was injected as the control for the remaining mice. Caliper measurements were made of the tumor diameters, and the tumor volume was calculated using the following formula: 0.5 × length × width^2^. Tumor volumes and mouse body weights were measured every 2 days. When the tumor volume reached 2000 mm^3^ the animals were killed. Then tumors were excised and weighed.

### 2.16 Statistical analysis

All data are expressed as mean ± SD and are representative of at least three separate experiments. Student’s *t* test was used to compare two independent groups, and the corresponding bar graph or line charts were drawn using GraphPad Prism 7 software. The differences between continuous variables were using the unpaired *t* test or Mann‐Whitney *U* test. Probability values less than .05 indicated statistical significance.

## 3 RESULTS

### 3.1 Elevated PDK4 expression is associated with R‐CHOP resistance in DLBCL cells

**FIGURE 1:**
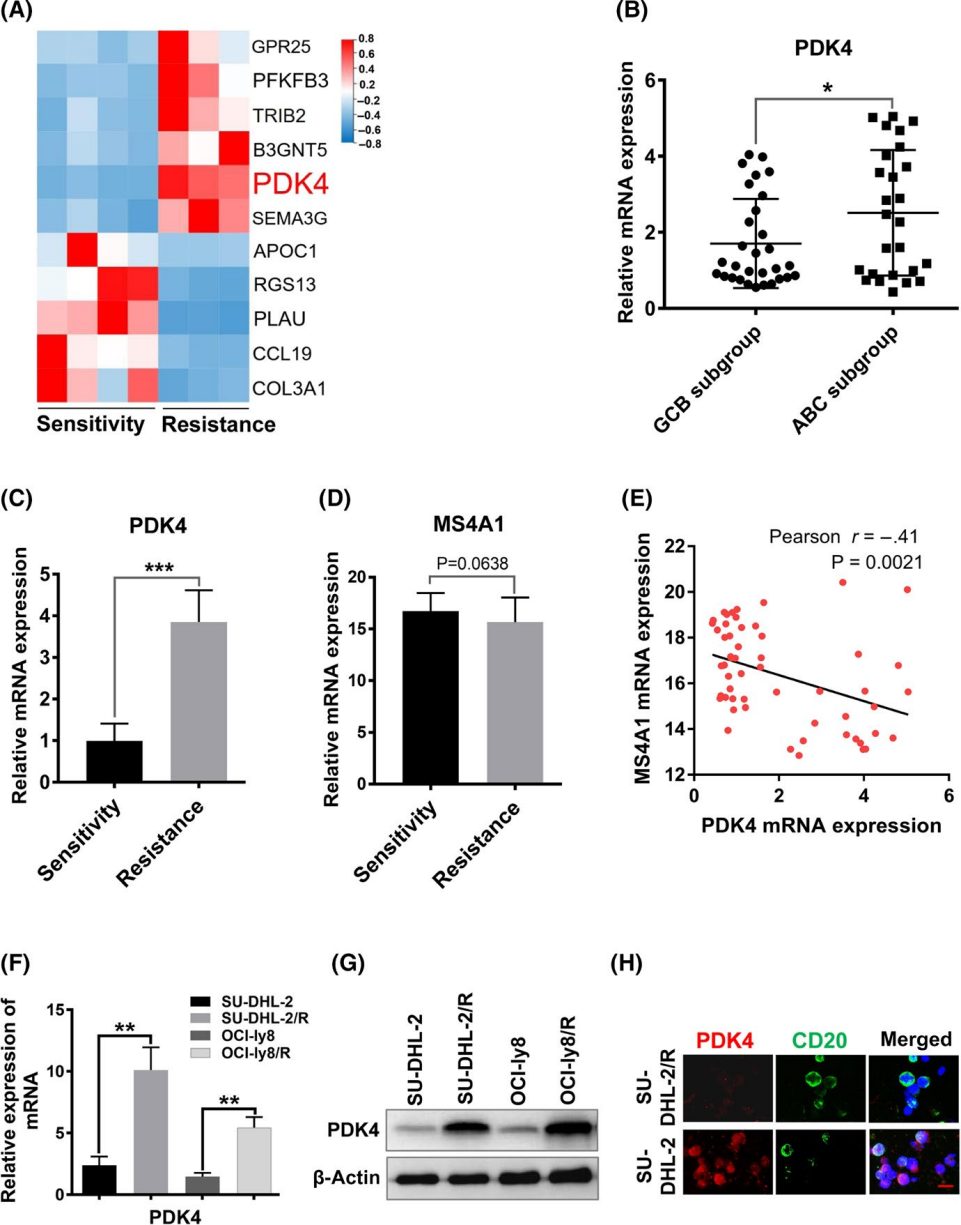
High pyruvate dehydrogenase kinase 4 (PDK4) is associated with R‐CHOP (rituximab plus cyclophosphamide, doxorubicin, vincristine, and prednisone) resistance in diffuse large B‐cell lymphoma (DLBCL) cells. A, Gene expression detected by RNA sequencing and expression of PDK4 in DLBCL patients. Hierarchical cluster analysis of the top 11 deregulated genes in R‐CHOP‐sensitive patients (n = 4) and R‐CHOP‐resistant patients (n = 3). Upregulated genes are shown in red and downregulated genes are shown in blue. B, PDK4 mRNA expression between germinal‐center B‐cell‐like (GCB)‐DLBCL (n = 30) and activated B‐cell‐like (ABC)‐DLCBL (n = 26) subtypes among 56 cases in the DLBCL cohort. *P < .05. C, D, Real‐time quantitative PCR analysis of PDK4 mRNA and MS4A1 mRNA expression in R‐CHOP‐sensitive patients (n = 37) and R‐CHOP‐resistant patients (n = 19) with DLBCL. E, Pearson correlation analysis for PDK4 and MS4A1 expression in 56 cases in the DLBCL cohort (Pearson *r* = −0.41, *P* = .0021, with *F* test). F, G, Assessment of mRNA and protein levels of PDK4 in R‐CHOP‐resistant DLBCL cell line SU‐DHL‐2/R and rituximab‐resistant DLBCL cell line OCI‐ly8/R, as well as their parental cell lines. H, Representative images of immunofluorescence analysis for PDK4 (red) and CD20 (green) protein expression in SU‐DHL‐2/R and parental cells. Scale bar, 15 μm

The clinical characteristics of patients are detailed in Table S2. All patients were treated with the R‐CHOP regimen in the primary therapy. Responses to treatment were evaluated by CT scans or PET/CT following the response criteria for lymphoma.^40^ Patients with DLBCL treated with R‐CHOP regimen were divided into sensitive (n = 37) and resistant (n = 19) groups according to treatment response. Resistant patients were defined as failing to achieve complete remission or developing rapid disease progression (less than 6 months) after six to eight cycles of R‐CHOP treatment. We first screened the DEGs between four R‐CHOP‐sensitive patients and three R‐CHOP‐resistant patients using RNA sequencing analysis. The results indicated the expression of PDK4 was markedly elevated in resistant patients compared with sensitive patients (Figure 1A and Table S3). Moreover, higher PDK4 expression was observed in patients in the ABC subgroup (n = 26) than in patients in the GCB subgroup (n = 30) of DLBCL (*P* = .037; Figure 1B). Consistently, among the 56 patients we found that PDK4 mRNA expression presented significant difference with higher levels in resistant patients (*P* < .001; Figure 1C). We further investigated the MS4A1 mRNA expression levels in the two groups. The data did not show significant difference between sensitive patients and resistant patients (*P* = .063; Figure 1D). However, an inverse correlation between PDK4 expression and MS4A1 expression was observed using Pearson’s correlation analysis (*r* = −.41, *P* = .0021; Figure 1E). In addition, the R‐CHOP‐resistant DLBCL cell line SU‐DHL‐2/R showed higher PDK4 than the R‐CHOP‐sensitive DLBCL cell line SU‐DHL‐2 at both the mRNA and protein levels, as well as rituximab‐resistant DLBCL cell line OCI‐ly8/R compared with rituximab‐sensitive DLBCL cell line OCI‐ly8 (Figure 1F,G). The drug resistance abilities of the R‐CHOP‐resistant and rituximab‐resistant DLBCL cell lines were identified by CCK‐8 assay (Figure S1). Subsequently, immunofluorescence was used to detect the expression of PDK4 and CD20 in R‐CHOP‐resistant DLBCL cells. As a result, the expression of PDK4 was elevated in SU‐DHL‐2/R compared with the parental cells, and SU‐DHL‐2/R cells showed downregulated CD20 expression (Figure 1H). These findings indicate that PDK4 plays an important role in R‐CHOP and rituximab resistance.

### 3.2 Pyruvate dehydrogenase kinase 4 is associated with MS4A1/CD20 level and rituximab sensitivity in DLBCL cells

**FIGURE 2:**
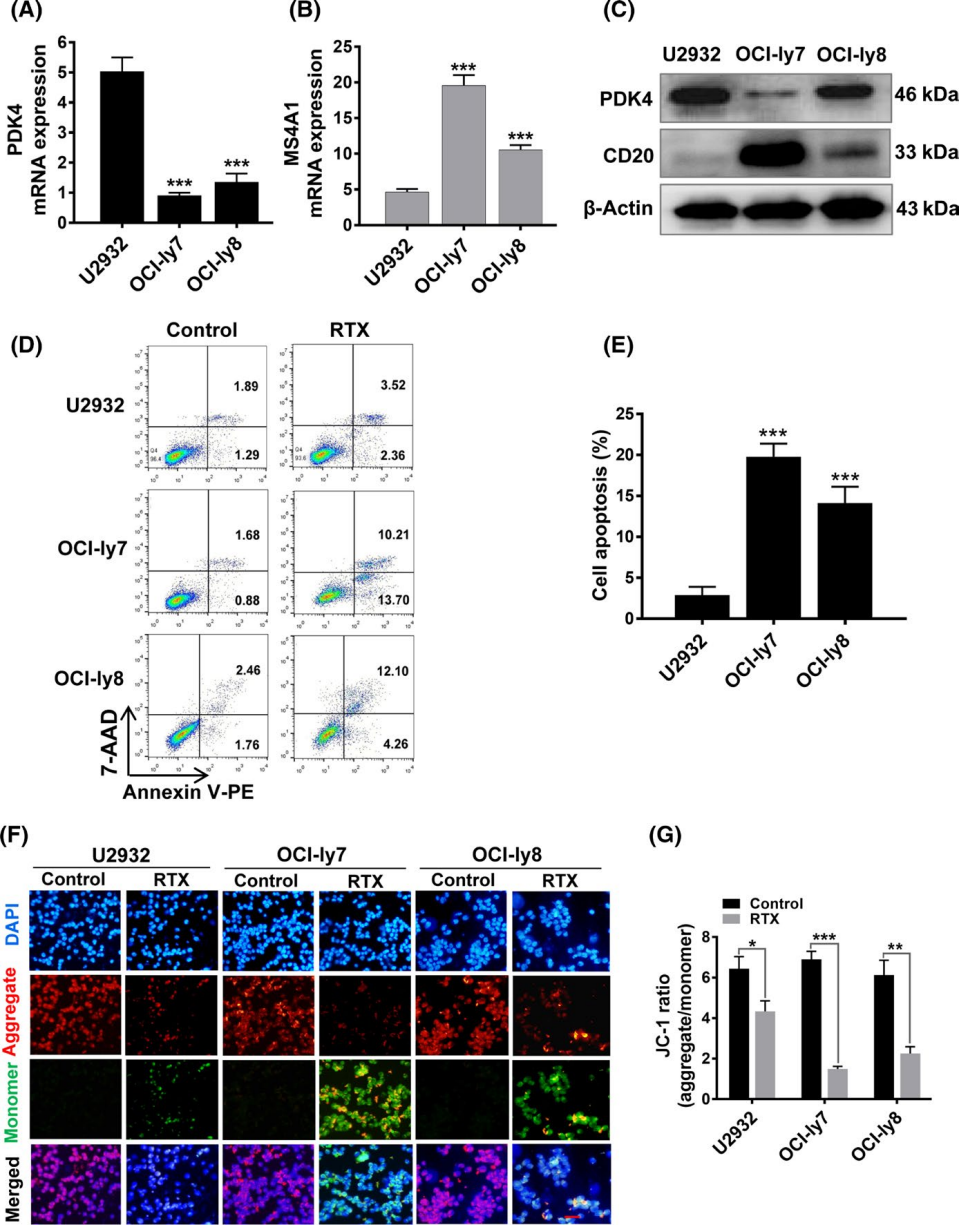
High pyruvate dehydrogenase kinase 4 (PDK4) is associated with rituximab (RTX) resistance and low MS4A1/CD20 in diffuse large B‐cell lymphoma (DLBCL) cells. A, B, Real‐time quantitative PCR analysis of PDK4 and MS4A1 mRNA expression in DLBCL cell lines U2932, OCI‐ly7, and OCI‐ly8. C, Western blot analysis of PDK4 and CD20 protein levels in DLBCL cell lines U2932, OCI‐ly7, and OCI‐ly8. D, E, Annexin V‐phycoerythrin (PE)/7‐AAD double staining analysis of the three DLBCL cell lines treated with RTX (50 μg/mL). F, G, Mitochondrial membrane potential of DLBCL cells following treatment with RTX for 48 hours and stained with JC‐1 probe. Representative pictures of JC‐1 staining are shown. Scale bar, 25 μm. ***P < .001

In order to investigate the potential roles of PDK4 expression in rituximab resistance, we used three DLBCL cell lines, U2932, OCI‐ly7, and OCI‐ly8, for in vitro assays, including qRT‐PCR, flow cytometry, and western blotting assays. Good concordance was observed between mRNA and protein expression for PDK4, with U2932 showing the highest and OCI‐ly7 showing the lowest expression (Figure 2A,C). Consistently, obvious inverse correlation between PDK4 expression and MS4A1/CD20 expression (Figure 2A‐C) was observed in these DLBCL cell lines. When these cell lines were treated with rituximab (50 μg/mL) for 48 hours, we observed a negative relationship between PDK4 expression and rituximab sensitivity, which depended on the expression levels of MS4A1/CD20.^41^, ^42^ As shown in Figure 2D,E, cell lines with low expression of PDK4 (OCI‐ly7 and OCI‐ly8) produced significant apoptosis (*P* < .001) compared with the high expression cell line (U2932). Additionally, by using JC‐1 dye staining we observed that the ratio of red / green signals in PDK4^low^ cells (OCI‐ly7 and OCI‐ly8) decreased more than that in PDK4^high^ cells (U2932) after treatment with rituximab (Figure 2F,G). The decreased ratio of red / green signals indicates mitochondrial damage and cell apoptosis.

### 3.3 Targeting PDK4 increases rituximab sensitivity against DLBCL cells

**FIGURE 3:**
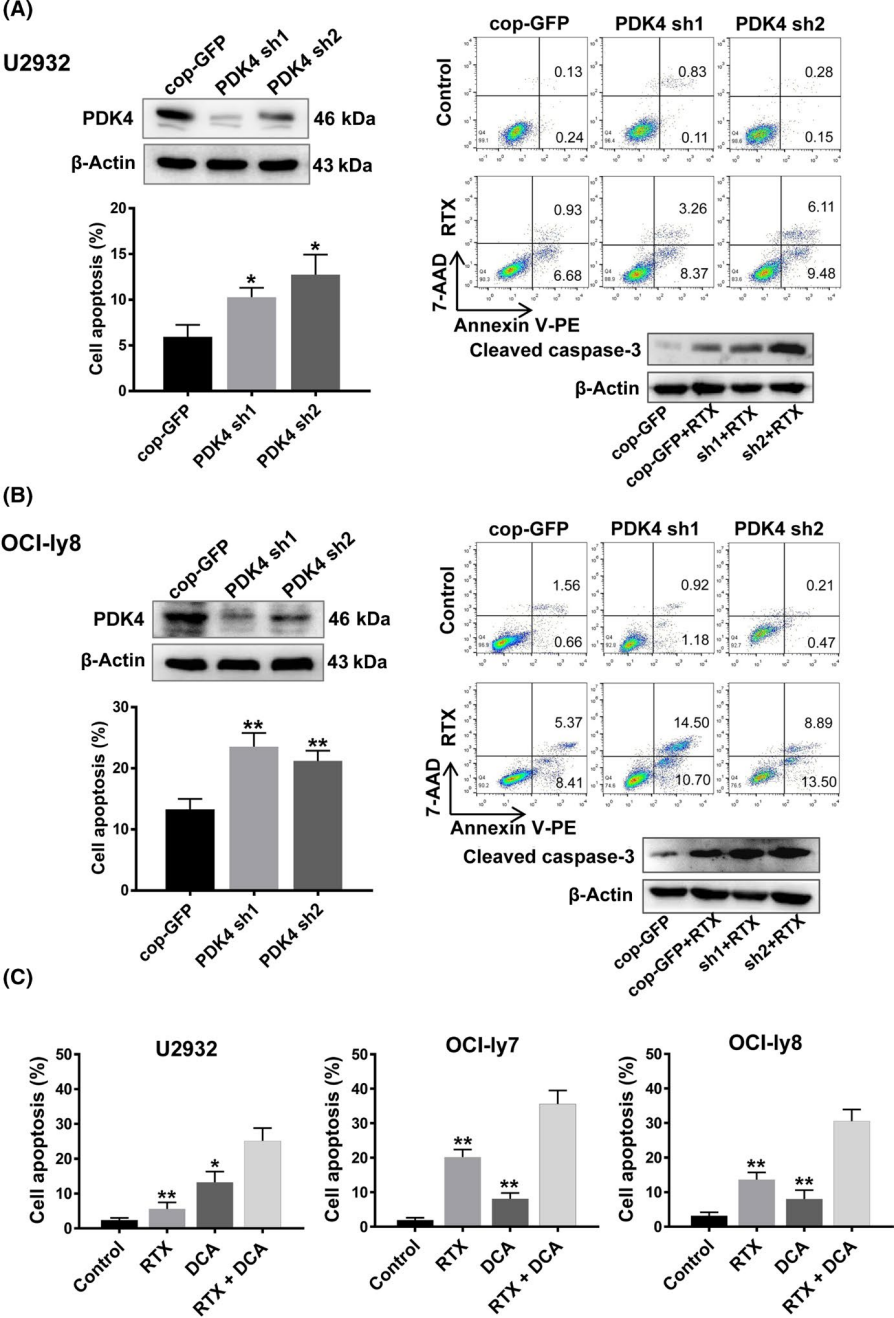
Inhibition of pyruvate dehydrogenase kinase 4 (PDK4) by shRNA or dichloroacetate (DCA) sensitizes diffuse large B‐cell lymphoma (DLBCL) cells to rituximab (RTX). A, B, Annexin V‐phycoerythrin (PE)/7‐AAD double staining analysis of DLBCL cells treated with RTX (50 μg/mL). Interference with PDK4 shRNA increased RTX‐induced cell apoptosis and caspase‐3 activation in U2932 and OCI‐Ly8 cell lines. C, PDK4 inhibitor dichloroacetate (DCA) enhanced the RTX‐induced apoptosis in DLBCL cell lines U2932, OCI‐ly7, and OCI‐Ly8. cop‐GFP, copepod super green fluorescent protein. *P < .05, **P < .01

To explore the effect of PDK4 on cell growth and rituximab resistance in DLBCL cells, two shRNA sequences (shRNA1 and shRNA2) targeting human PDK4 were designed. We generated PDK4‐deficient stable cell lines using shRNAs (PDK4 sh1 and PDK4 sh2) in U2932 and OCI‐ly8 cell lines, which resulted in significant loss of PDK4 protein expression, and observed significant increase in the percentage of apoptosis and caspase‐3 activation in transduced cells after rituximab treatment (U2932 PDK4 sh1, *P* = .012; U2932 PDK4 sh2, *P* = .011; and OCI‐ly8 PDK4 sh1, *P* = .0032; OCI‐ly8 PDK4 sh2, *P* = .0045; Figure 3A,B).

Dichloroacetate, a specific inhibitor of PDK4,^43^, ^44^ was used to evaluate the effect of pharmacological PDK4 inhibition. We observed a significant increase of apoptosis in cell lines when treated with rituximab (50 μg/mL) and DCA (5 mmol/L) for 48 hours compared with rituximab alone (OCI‐ly7, *P* = .0038; OCI‐ly8, *P* = .0018; Figure 3C), and even in the U2932 cell line, which initially showed poor response to rituximab (*P* = .0012; Figure 3C). These data suggested that the PDK4 inhibitor DCA can effectively reverse rituximab resistance in DLBCL cells.

### 3.4 Pyruvate dehydrogenase kinase 4 has a negative regulatory effect on MS4A1/CD20 expression in DLBCL cells

**FIGURE 4:**
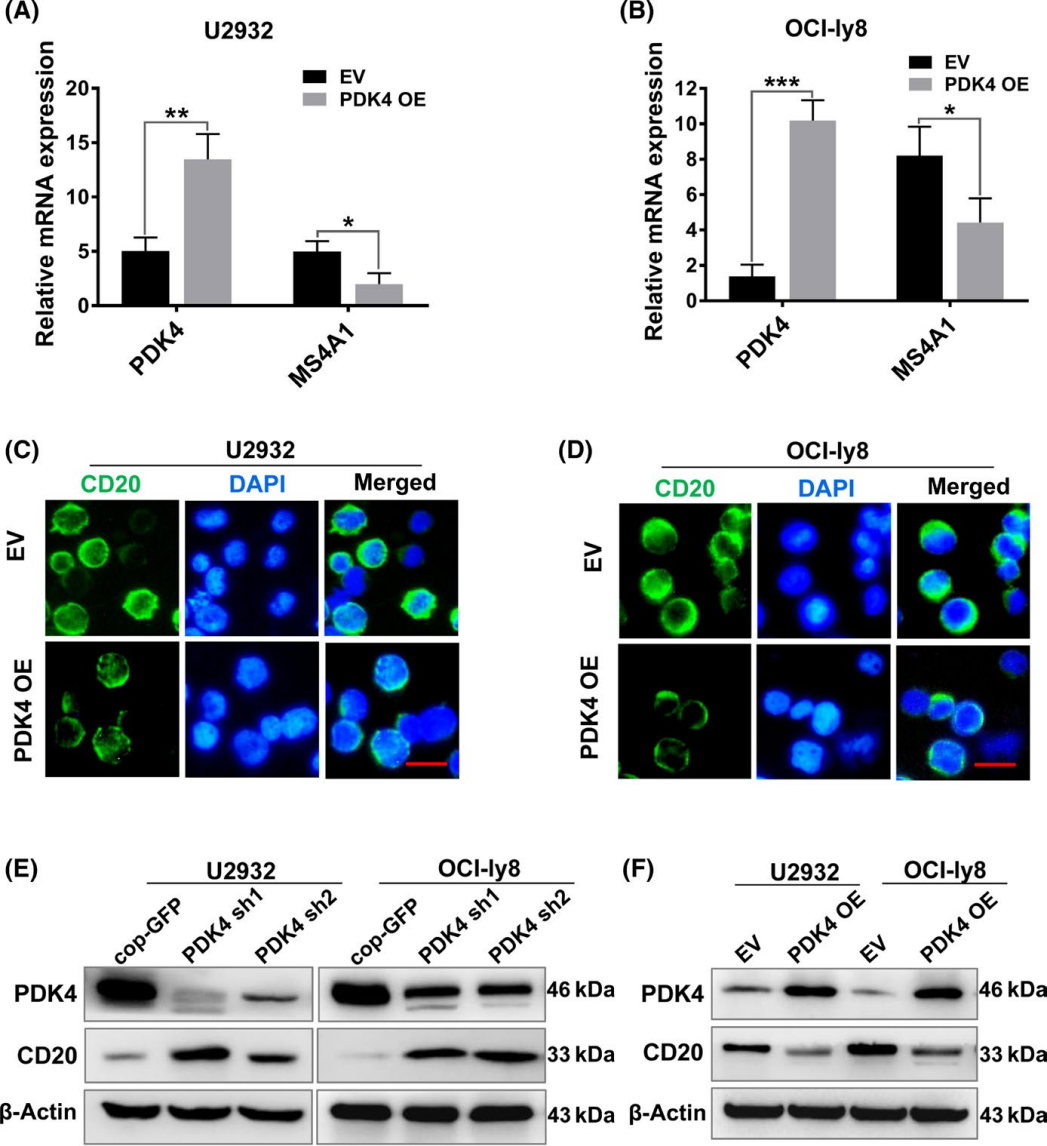
Pyruvate dehydrogenase kinase 4 (PDK4) has a negative regulatory effect on MS4A1/CD20 expression in diffuse large B‐cell lymphoma cells. A, B, Real‐time quantitative PCR analysis of PDK4 and MS4A1 mRNA expression in U2932 and OCI‐Ly8 cells transfected with PDK4 overexpressing (PDK4 OE) plasmid or empty vector (EV). C, D, CD20 (green) molecules in the plasma membrane showed a reduction after PDK4 overexpression in both U2932 and OCI‐Ly8 cells. Scale bar, 15 μm. E, F, Western blot analysis of PDK4 and CD20 protein levels in U2932 and OCI‐Ly8 cells with PDK4 shRNA interference or transfected with PDK4 OE plasmid or EV. *P < .05, **P < .01, ***P < .001

Pyruvate dehydrogenase kinase 4 functions as a positive regulator of glycolysis during tumor development.^25^ Rituximab carries out its action through ligation with the cell surface CD20 molecule.^45^ To further determine the regulatory effect of PDK4 on MS4A1/CD20 in DLBCL cells, we overexpressed PDK4 by lentivirus in both U2932 and OCI‐ly8 cell lines (Figure 4A,B,F). As shown in Figure 4A,B, qRT‐PCR analysis confirmed that when PDK4 was overexpressed in U2932 and OCI‐ly8 cells (*P* < .01), this led to a corresponding reduction in MS4A1 mRNA levels (*P* < .05). Using confocal microscopy, we observed that CD20 molecules located in the plasma membrane showed a remarkable reduction after overexpressing PDK4 (PDK4 OE) compared with the control (EV) in both U2932 and OCI‐ly8 cells (Figure 4C,D). Furthermore, protein levels of CD20 were increased at deletion of PDK4 by shRNA (Figure 4E) and decreased at overexpression of PDK4 by lentivirus (Figure 4F). Moreover, our results showed that PDK4 knockdown significantly increased rituximab sensitivity that relies on MS4A1/CD20 expression levels.^41^, ^42^ Collectively, these results indicate that PDK4 plays a negative role in regulation of MS4A1/CD20 expression in DLBCL cells.

### 3.5 Rituximab‐resistant DLBCL cells show a metabolic shift of active glycolysis and OXPHOS

**FIGURE 5:**
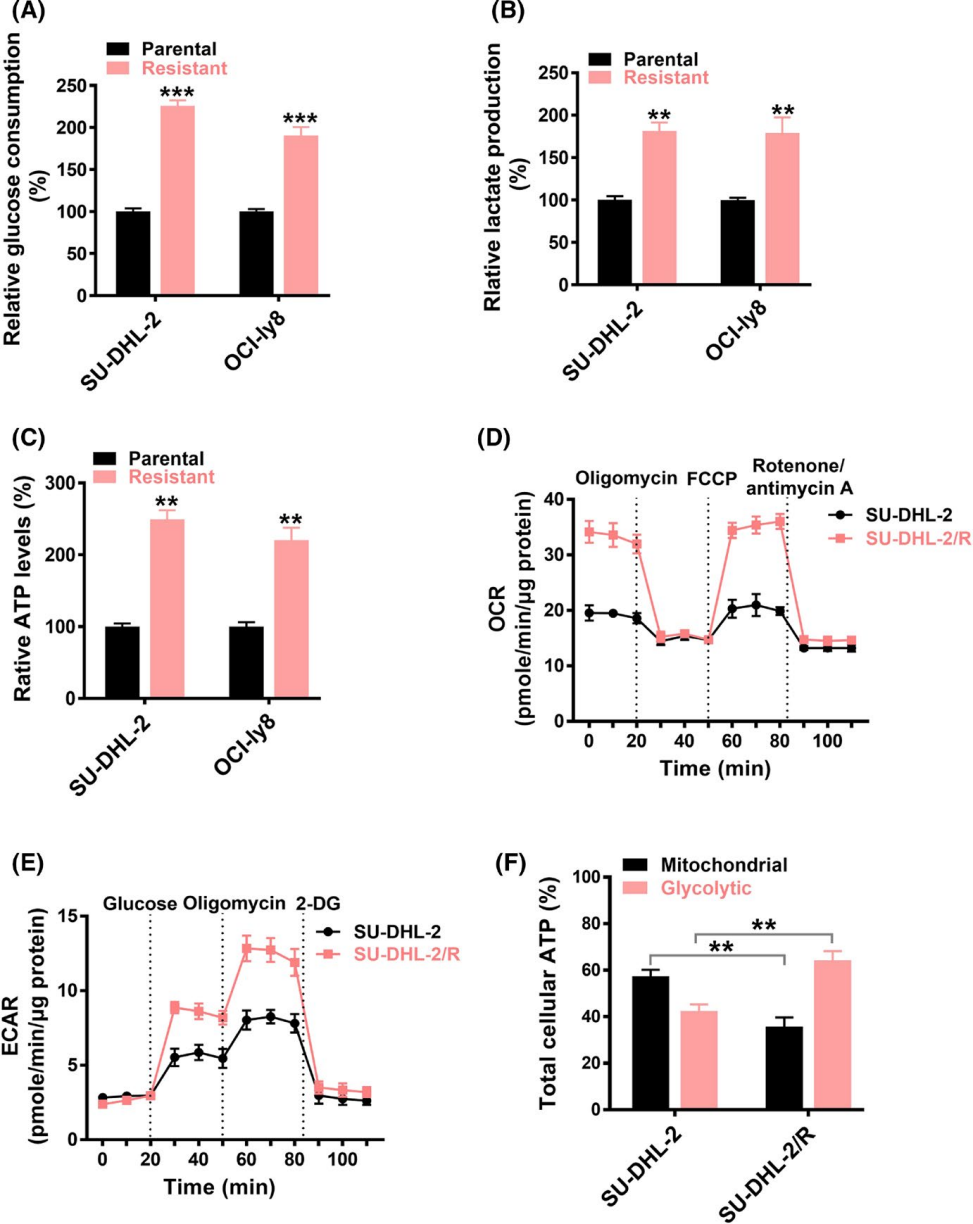
Rituximab‐resistant diffuse large B‐cell lymphoma (DLBCL) cells show a metabolic shift of active glycolysis and oxidative phosphorylation (OXPHOS). A‐C, Glucose consumption (A), lactate production (B), and ATP levels (C) in R‐CHOP (rituximab plus cyclophosphamide, doxorubicin, vincristine, and prednisone)‐resistant DLBCL cell line SU‐DHL‐2/R and rituximab‐resistant DLBCL cell line OCI‐ly8/R, as well as their parental cell lines. D, E, Cellular oxygen consumption rate (OCR) (D) or extracellular acidification rate (ECAR) (E) of SU‐DHL‐2/R and SU‐DHL‐2 cells was determined. F, Percent contribution of glycolysis and mitochondrial metabolism to total cellular ATP of SU‐DHL‐2/R and parental cells according to energy budget calculations. FCCP, carbonyl cyanide p‐trifluoromethoxyphenylhydrazone. **P < .01, ***P < .001

Previous studies have identified that there is metabolic heterogeneity in DLBCL.^9^, ^10^ We therefore determined the metabolic profiles of SU‐DHL‐2/R and OCI‐ly8/R cells. Our data showed that the SU‐DHL‐2/R and OCI‐ly8/R cells showed significant increased glucose consumption (Figure 5A) and lactate production (Figure 5B) rates than their parental cells. Moreover, the extracellular ATP levels in SU‐DHL‐2/R and OCI‐ly8/R cells were significantly higher than in their parental cells (Figure 5C).

Cells produce ATP through mitochondrial OXPHOS and glycolysis. To determine which process was involved in the rituximab‐resistant cells, cellular OXPHOS and glycolysis were monitored by measuring the OCR and ECAR in real time. As suggested by the results, SU‐DHL‐2/R cells showed a significant increase in cellular OCR (Figure 5D), indicating an increase in the amount of ATP produced from mitochondrial OXPHOS in resistant cells compared to the parental cells. Seahorse analysis showed that the ECAR of SU‐DHL‐2/R cells was higher than that of the parental cells (Figure 5E), reflecting the increase of total glycolytic flux. To compare mitochondrial OXPHOS and glycolysis in their contribution to the cellular energy budget, the proportion of total cellular ATP was assessed.^9^ Compared with the parental cells, SU‐DHL‐2/R cells accounted for a significantly higher proportion of its total energy from glycolysis (~65%) than from mitochondrial oxidative metabolism (Figure 5F). All these data suggested that the rituximab‐resistant cells showed increased ATP production and glycolysis compared to their parental cells.

### 3.6 Pyruvate dehydrogenase kinase 4 mediates the metabolic shift of rituximab‐resistant DLBCL cells

**FIGURE 6:**
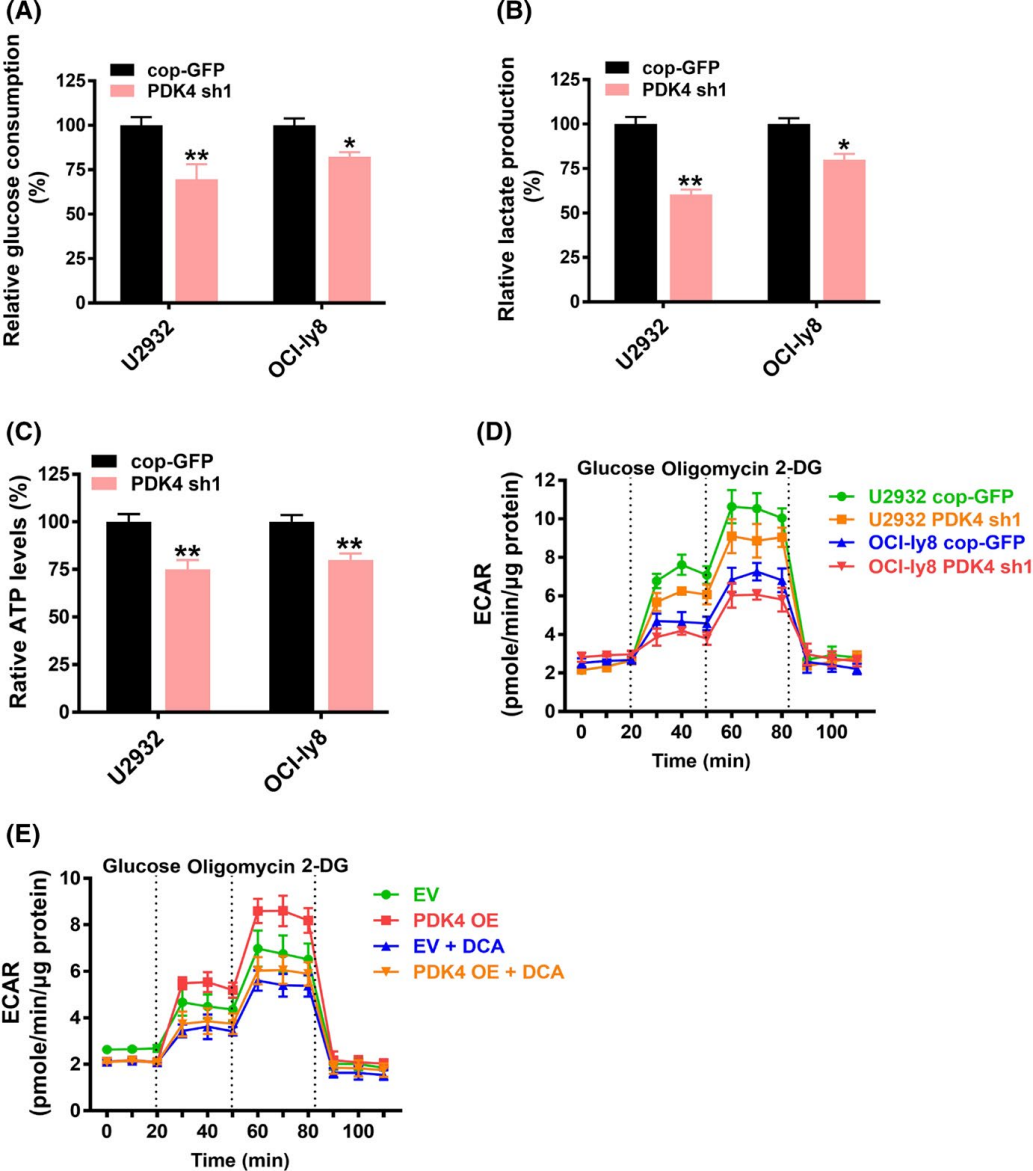
Pyruvate dehydrogenase kinase 4 (PDK4) mediates the metabolic shift of rituximab‐resistant diffuse large B‐cell lymphoma cells. A‐D, U2932 and OCI‐ly8 cells were transfected with cop‐GFP or interfered PDK4 shRNA (PDK4‐sh1) and the glucose consumption (A), lactate production (B), ATP levels (C), and cellular extracellular acidification rate (ECAR) (D) were measured. E, OCI‐Ly8 cells were transfected with PDK4 overexpressing (PDK4 OE) plasmid or empty vector (EV). After treatment with or without dichloroacetate (DCA, 5 mM), cellular ECAR were measured. cop‐GFP, copepod super green fluorescent protein. *P < .05, **P < .01

We further investigated whether PDK4 was involved in the metabolic shift of rituximab‐resistant DLBCL cells. The results showed that the glucose consumption (Figure 6A) and lactate production (Figure 6B) of PDK4‐deficient (PDK4 sh1) U2932 and OCI‐ly8 cells were obviously decreased compared with that in control cells. Consistently, the extracellular ATP levels of PDK4‐deficient cells were also reduced compared to control cells (Figure 6C). Seahorse analysis showed that the ECAR of PDK4‐deficient cells was decreased (Figure 6D). However, the effects of PDK4 deficiency on metabolic features were weaker in OCI‐ly8 cells than in U2932 cells (Figure 6A‐D), which might be due to rituximab‐resistant cells being more reliant on glycolysis. In addition, the ECAR of PDK4 OE cells was markedly increased. However, this was reversed after treatment with PDK4 inhibitor DCA (Figure 6E). These results suggested that PDK4 regulates the metabolic shift of rituximab‐resistant DLBCL cells.

### 3.7 Overexpression of PDK4 promotes proliferation and rituximab resistance in DLBCL cells

**FIGURE 7:**
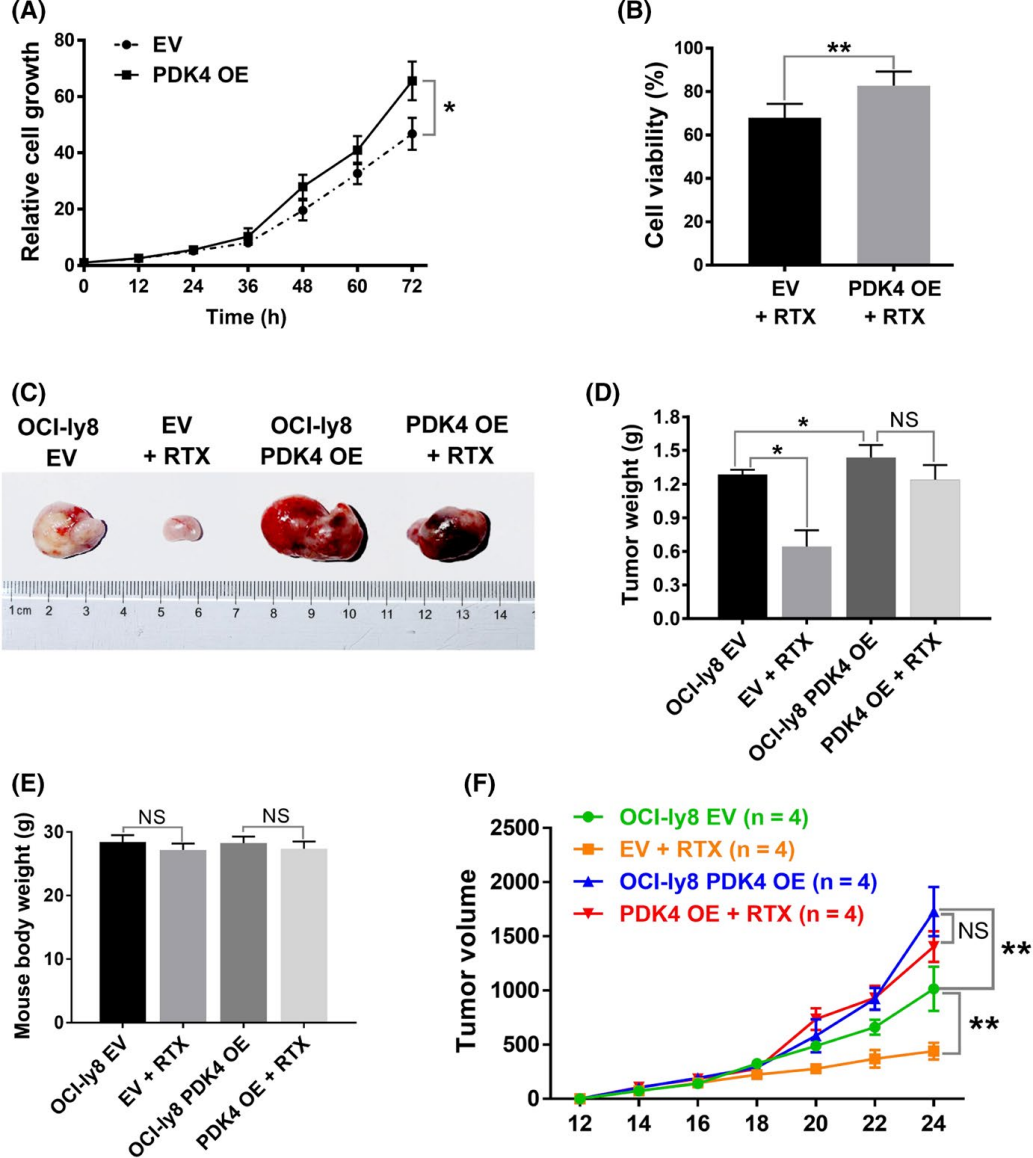
Overexpression of pyruvate dehydrogenase kinase 4 (PDK4) promotes proliferation and rituximab resistance in diffuse large B‐cell lymphoma cells. A, In vitro cell growth assay showing relative cell growth curves of OCI‐Ly8 empty vector (EV) and OCI‐Ly8 PDK4 overexpressing (OE) cells treated with rituximab (RTX, 50 μg/mL) for indicated times. B, OCI‐Ly8 EV and PDK4 OE cells were treated with RTX for 48 hours, and cell viability rates were analyzed using CCK‐8 assays. C, D, F, in vivo xenograft mouse models of OCI‐ly8 PDK4 OE or OCI‐ly8 EV cells treated with RTX (12.5 mg/kg) or PBS. Differences in tumor weight (D) and tumor volume (F) are shown between those four groups (n = 4). E, Body weight of mice in each experimental group measured at day 24. NS *P* *> .05*, *P < .05, **P < .01

To ascertain whether high expression of PDK4 can promote cell growth and enhance rituximab resistance, a further study was carried out using the PDK4 OE DLBCL cell lines U2932 and OCI‐ly8. As shown in Figure 7A, the capabilities of cell growth were significantly increased for PDK4 OE OCI‐ly8 cells compared to controls after treatment with rituximab (50 μg/mL) for 72 hours (*P* = .022). Subsequently, CCK‐8 assay was carried out using the PDK4 OE U2932 and OCI‐ly8 cells treated either with or without rituximab. U2932 (Figure S2) and OCI‐ly8 PDK4 OE cells (Figure 7B) showed a sharp increase in proliferative activity when compared with controls.

The above results have confirmed that knockdown of PDK4 sensitizes DLBCL cells to rituximab in vitro. To further confirm our conclusion, we asked whether OE of PDK4 promotes rituximab resistance in DLBCL cells in vivo. We prepared a xenograft mouse model of DLBCL by subcutaneously transplanting PDK4 OE OCI‐ly8 cells into immunocompromised B‐NDG mice; B‐NDG mice transplanted with the same number of OCI‐ly8 cells transfected with EV were used as controls. At 10 days after the injection of DLBCL cells, half of the mice derived from both the PDK4 OE group and EV group were treated with rituximab (12.5 mg/kg) injected intraperitoneally daily for 2 weeks. As shown in Figure 7C,D,F, the PDK4 OE tumors that developed in untreated mice were larger than the EV tumors (tumor weight, 0.042; tumor volume, *P* = .0034). There was no difference in mouse body weight, indicating rituximab treatment had no obvious toxicity (Figure 7E). In addition, xenografts with EV showed significant tumor shrinkage after treatment with rituximab in terms of tumor weight (*P* = .029) and tumor volume (*P* = .0019), whereas those with PDK4 OE did not have significantly reduced tumor weight (*P* = .158) or tumor volume (*P* = .083).

## 4 DISCUSSION

**FIGURE 8:**
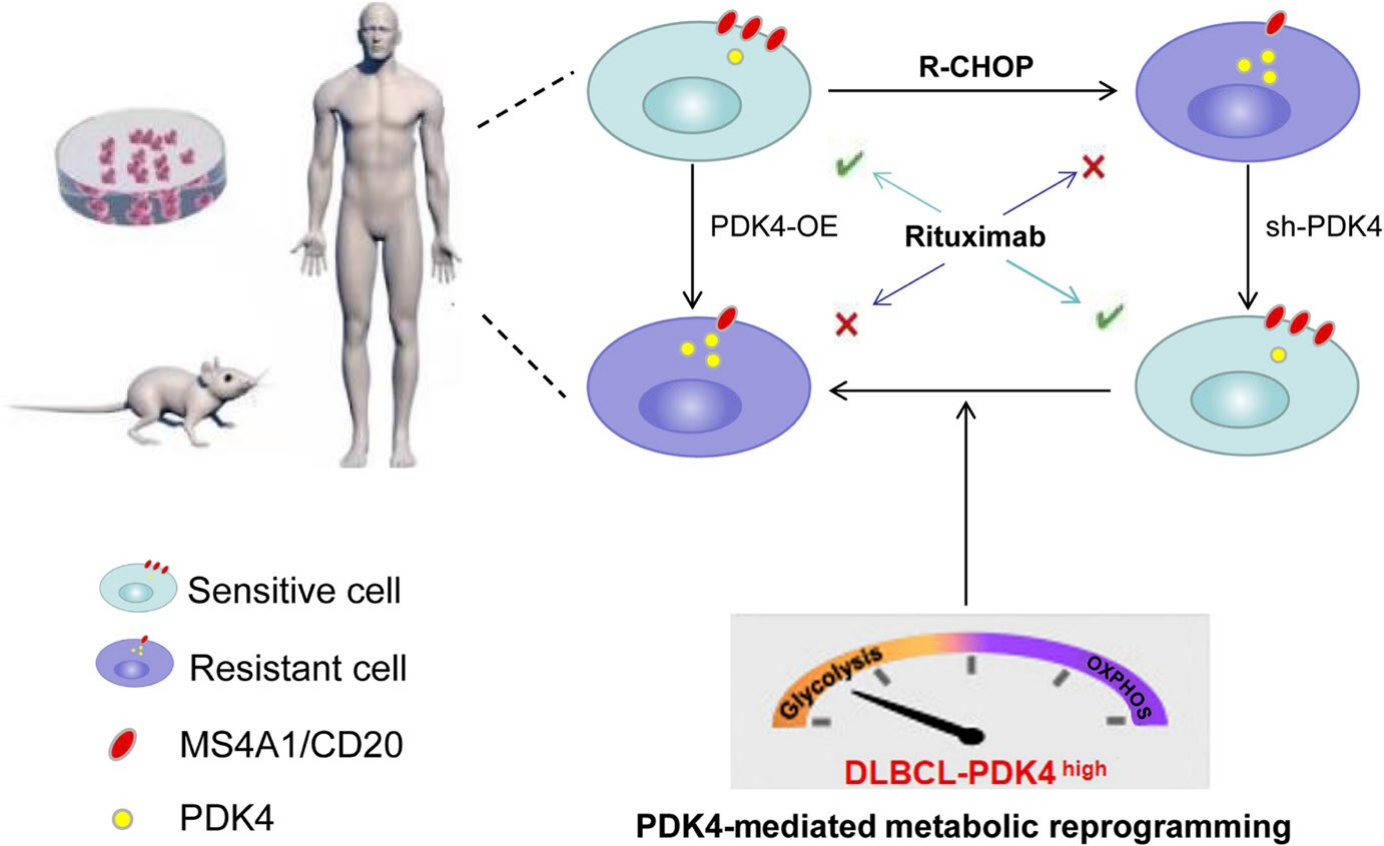
Model of our working hypothesis of rituximab resistance in diffuse large B‐cell lymphoma cells. OE, overexpressing; PDK4, pyruvate dehydrogenase kinase 4; R‐CHOP, rituximab plus cyclophosphamide, doxorubicin, vincristine, and prednisone

Metabolic reprogramming has been considered as a key marker of cancer progression, as well as being involved in drug resistance.^20^, ^46^ In the present study, we found that PDK4 was overexpressed in rituximab‐resistant DLBCL cells. We further showed that PDK4 promotes cell growth and rituximab resistance by mediating metabolic shift in DLBCL cells. Importantly, our data suggested that PDK4 promotes rituximab resistance, at least in part, by regulating MS4A1/CD20 expression (Figure 8).

Previous studies have indicated that suppression of PDK4 inhibits cell proliferation, increases apoptosis, and regulates sensitivity of drugs in solid tumors.^20^, ^22^, ^47^, ^48^ Moreover, PDK4 promotes tumor progression in lung, cervical, and liver cancer.^20^, ^25^, ^48^, ^49^ Therefore, PDK4 could act as an oncogene in cancer. However, the functional role of PDK4 in DLBCL remains unclear. In this study, we showed that loss of PDK4 expression or PDK4 inhibitor treatments can effectively enhance rituximab‐induced apoptosis in DLBCL cells. The effects of PDK4 on DLBCL cell growth and rituximab sensitivity were further supported by our in vitro and in vivo data that PDK4 OE tumors grow faster and show less response to treatment with rituximab. The above data suggested that PDK4 promotes rituximab resistance in DLBCL cells, and PDK4 could be a potential target for DLBCL therapy.

Acquirement of resistance to rituximab and down‐modulation of CD20 expression after rituximab therapy have been observed in DLBCL patients.^17^, ^18^ Repeated exposure to rituximab led to a gradual reduction of CD20 expression during the development of rituximab‐resistant cell lines.^18^ Recent reports suggest that a CD20‐negative phenotypic change occurs in a certain number (approximately 26.3%) of CD20‐positive B‐cell lymphoma patients after rituximab‐based treatment.^17^ However, the mechanisms of CD20 loss remain unknown. Recent studies have shown that genetic and epigenetic mechanisms might be associated with low CD20 expression after rituximab treatment.^16^ Epigenetic therapies are able to restore both CD20 expression and rituximab sensitivity.^17^, ^19^ Any abnormality in the process of CD20 protein expression could lead to rituximab resistance, such as downregulation, or alterations in the cell membrane.^9^, ^14^, ^29^ In this study, we found that PDK4 could perform a critical role in MS4A1/CD20 expression, because lowered CD20 mRNA and protein levels were restored in cells with PDK4 shRNAs and the mRNA and protein level of CD20 was attenuated after PDK4 overexpression in DLBCL cells. In addition, we observed a negative relationship between PDK4 expression and MS4A1/CD20. These results indicated that PDK4 has a negative regulatory effect on MS4A1/CD20 expression in DLBCL cells.

Metabolic reprogramming involves the acceleration of glycolytic flux, high‐speed ATP production, and the accumulation of lactate, which contributes to tumor progression and resistance to cancer therapy.^50^ Tumor cells undergo metabolic alteration to fulfill the bioenergetic need for rapid cell proliferation.^46^ As an important mitochondrial matrix enzyme for cellular energy regulation,^51^ PDK4 inhibits the entry of pyruvate into the TCA cycle, thus switching energy derivation to cytoplasmic glycolysis rather than mitochondrial OXPHOS.^44^, ^52^ Recently, DLBCL has been identified as a metabolic heterogeneous disease.^9^ In this study, we found that PDK4 was significantly elevated in rituximab‐resistant DLBCL cells compared to sensitive cells, suggesting that PDK4 upregulation is associated with rituximab resistance. To better understand the role of PDK4 in rituximab resistance, several rituximab‐resistant cell lines were investigated and characterized. We observed a metabolic shift, in that the main energy source was changed from OXPHOS to glycolysis, as shown in metabolic studies. Furthermore, our data showed a metabolic signature of active glycolysis by upregulation of PDK4, indicating that PDK4‐mediated metabolic reprogramming is involved in regulating the glycolysis and rituximab sensitivity of DLBCL cells.

Recent studies showed that forced CD20 expression restored cytoplasmic but not surface CD20, suggesting the existence of a defect in CD20 protein transport in rituximab‐resistant cell lines.^18^ Therefore, our study provided conjecture from the perspective of metabolism, that PDK4‐mediated metabolic reprogramming might play a negative role in the transport of CD20 protein from cytoplasm to cytomembrane. A recent study identified that GAPDH, a metabolic regulatory enzyme, is associated with R‐CHOP sensitivity.^10^ Thus we can predict that PDK4 might have similar clinical significance in DLBCL. However, survival and prognosis analyses of the patients in this study were not performed and most patients were still in follow‐up. This would drive us to persist in this study in the future.

To summarize, this is the first study showing that modulating PDK4 can affect rituximab‐induced cell apoptosis on DLBCL. Notably, we identified the potential that PDK4 promotes proliferation and rituximab resistance in DLBCL cells by mediating metabolic reprogramming. Pyruvate dehydrogenase kinase 4 has a negative regulatory effect on MS4A1/CD20 expression in DLBCL cells. These findings indicate that targeting PDK4 has the potential to overcome rituximab resistance in DLBCL. Further studies on the relationship between PDK4‐mediated metabolic reprogramming and CD20 protein transport from cytoplasm to cytomembrane could lead to the discovery of novel mechanisms of MS4A1/CD20 downregulation in DLBCL.

## DISCLOSURE

The authors declare no competing financial interests.

## Supporting information

Supplementary Material
