# Supplementary material for: Pyruvate dehydrogenase kinase 4‐mediated metabolic reprogramming is involved in rituximab resistance in diffuse large B‐cell lymphoma by affecting the expression of MS4A1/CD20
Source: Cancer Sci. 2021 Jul 28;112(9):3585–97. doi: 10.1111/cas.15055 (PMC8409406; doi:10.1111/cas.15055)
Supplement: Supplementary file 1 — Supplementary Material [file CAS-112-3585-s001.doc]

**PDK4-mediated metabolic reprogramming is involved in rituximab resistance in DLBCL via affecting the expression of MS4A1/CD20**

Duanfeng Jiang^1,2^, Qiuyu Mo^2^, Xiaoying Sun^3^, Xiaotao Wang^2^, Min Dong^4^, Guozhen Zhang^2^, Fangping Chen^5^**^*^**, Qiangqiang Zhao^3,6^**^*^**

^1^ Department of Hematology, the Third Xiangya Hospital, Central South University, Changsha, 410013 China;

^2^ Department of Hematology, Affiliated Hospital of Guilin Medical University, Guilin, 541001 China;

^3^ Department of Hematology, The Qinghai Provincial People's Hospital, Xining, 810007 China.

^4^ Department of Hematology, The second Affiliated Hospital of Hainan Medical University, Haikou, 570311 China;

^5^ Department of Hematology, Xiangya Hospital, Central South University, Changsha, 410008 China;

^6^ Department of Blood Transfusion, the Third Xiangya Hospital, Central South University, Changsha, 410013 China

**Supplementary tables**

| **Table S1.** Primers used for the qRT-PCR analysis. | |
| --- | --- |
| Primer name | Sequence (5'→3') |
| PDK4-forward | CACGATGTGAATTGGTTGGT |
| PDK4-reverse | TGCCTTTGAGTGTTCAAGGA |
| MS4A1-forward | ATGAAAGGCCCTATTGCTATG |
| MS4A1-reverse | GCTGGTTCACAGTTGTATATG |
| β-actin-forward | GGACTTCGAGCAAGAGATGG |
| β-actin-reverse | AGCACTGTGTTGGCGTACAG |

| **Table S2.** Clinical characteristics of DLBCL patients (n = 56). | | | | | | |
| --- | --- | --- | --- | --- | --- | --- |
| Characteristics | Resistant group (n = 19) | |  | Sensitive group (n = 37) | | P value |
|  | n | % |  | n | % |  |
| Age (years) |  |  |  |  |  |  |
| Median | 41 |  |  | 45 |  |  |
| Range | 17-68 |  |  | 20-71 |  |  |
| >60 | 6 | 32 |  | 13 | 35 | 0.273 |
| Sex (male) | 10 | 53 |  | 19 | 51 | 0.532 |
| Performance status (ECOG) > 1 | 3 | 16 |  | 5 | 14 | 0.360 |
| Ann Arbor stage > 2 | 12 | 63 |  | 18 | 49 | 0.095 |
| Extranodal sites > 1 | 4 | 21 |  | 5 | 14 | 0.118 |
| Hans criteria |  |  |  |  |  | 0.013 |
| GCB | 7 | 37 |  | 23 | 62 |  |
| Non‐GCB | 12 | 63 |  | 14 | 38 |  |
| IPI risk group |  |  |  |  |  | 0.109 |
| Low (0‐2) | 13 | 68 |  | 33 | 89 |  |
| High (3‐5) | 6 | 32 |  | 4 | 11 |  |
| LDH > normal | 9 | 47 |  | 13 | 35 | 0.285 |
| HBV-DNA positive | 2 | 11 |  | 2 | 5 | 0.381 |

| **Table S3.** Eleven differentially expressed genes between R-CHOP sensitive and resistant patients. | | | | |
| --- | --- | --- | --- | --- |
| **Gene ID** | **Gene Symbol** | **log2FC** | **Qvalue** | **Up/down** |
| ENSG00000170128 | GPR25 | 2.012711104 | 1.91E-03 | Up |
| ENSG00000170525 | PFKFB3 | 3.374831484 | 1.25E-04 | Up |
| ENSG00000071575 | TRIB2 | 3.465485607 | 3.03E-06 | Up |
| ENSG00000176597 | B3GNT5 | 2.898737872 | 2.95E-02 | Up |
| ENSG00000004799 | PDK4 | 4.874503440 | 2.14E-11 | Up |
| ENSG00000010319 | SEMA3G | 4.286470945 | 3.42E-04 | Up |
| ENSG00000130208 | APOC1 | -2.137472247 | 5.65E-07 | Down |
| ENSG00000127074 | RGS13 | -2.785142328 | 1.97E-05 | Down |
| ENSG00000122861 | PLAU | -3.683254202 | 1.18E-04 | Down |
| ENSG00000172724 | CCL19 | -2.521137867 | 1.71E-05 | Down |
| ENSG00000168542 | COL3A1 | -2.861607442 | 2.92E-03 | Down |

**Supplementary figures**

**
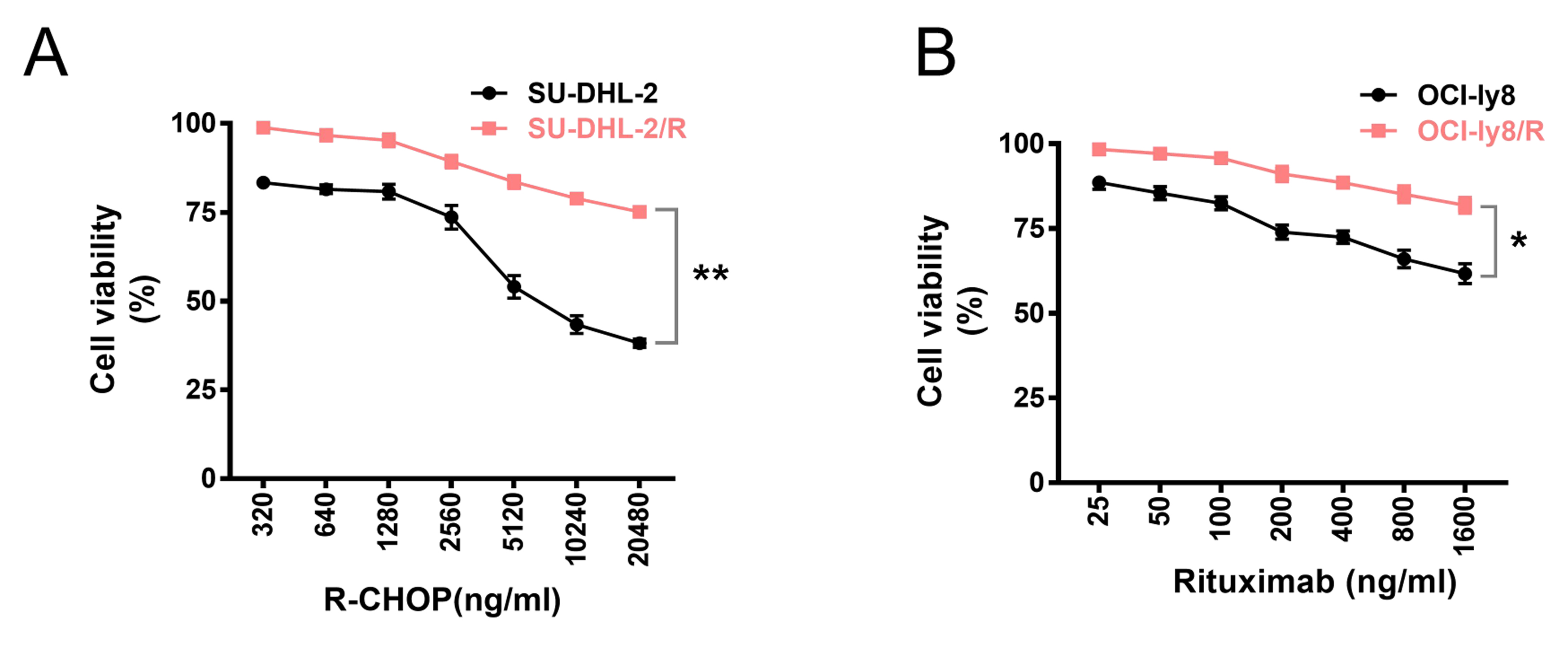
**

**Figure S1.** Identification of drug-resistant DLBCL cell lines using CCK-8 assays. A, SU-DHL-2/R cells resistant to R-CHOP compared with SU-DHL-2 cells (n = 6; ^**^ P < 0.01; with one-way ANOVA). B, OCI-ly8/R cells resistant to rituximab compared with OCI-ly8 cells (n = 6; ^*^ P < 0.05; with one-way ANOVA).


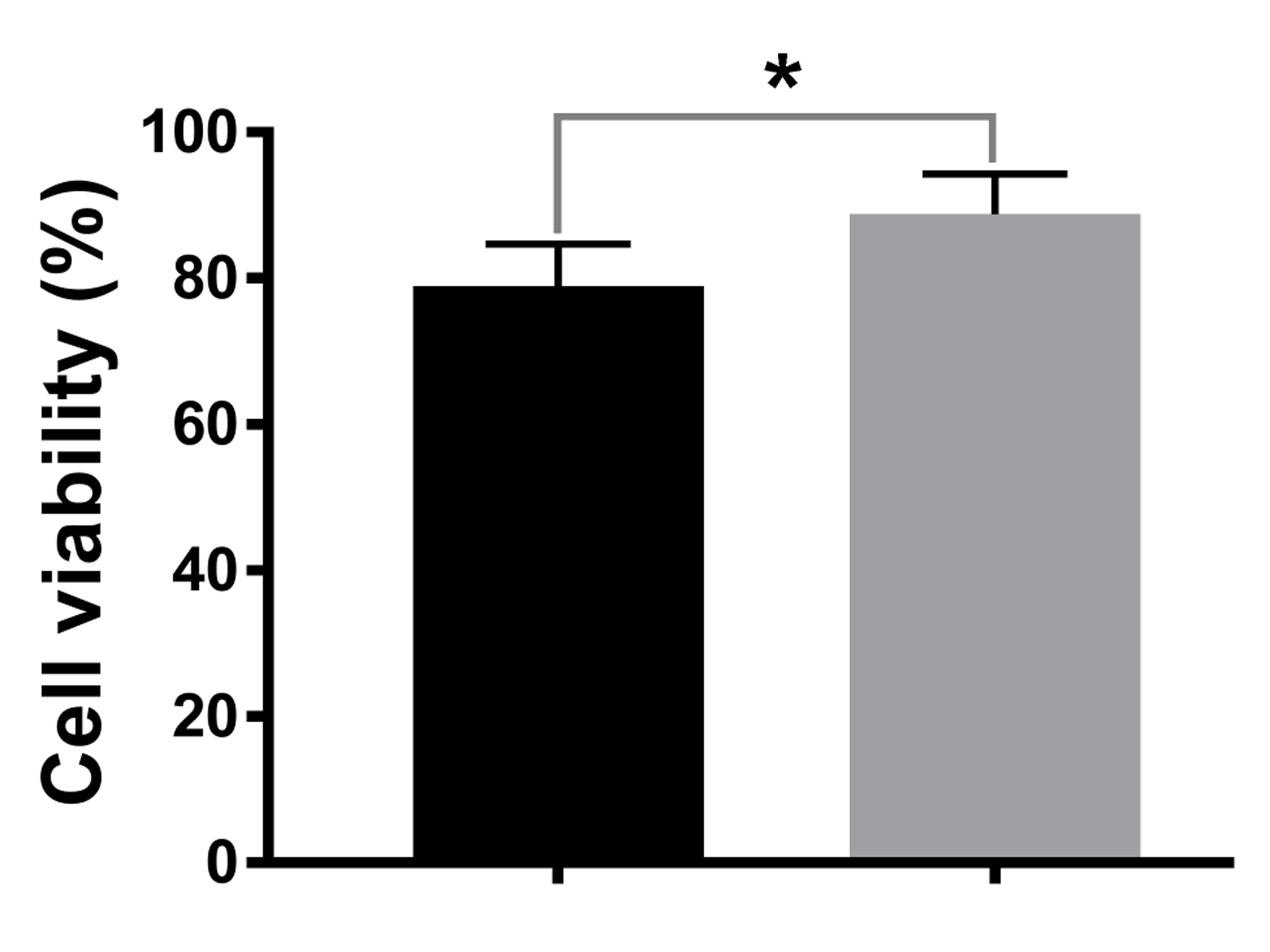


**Figure S2.** U2932 EV and PDK4 OE cells were treated with rituximab (50 μg/ml) for 48 hours, the cell viability rate were analysed using CCK-8 assays (n = 6, * P < 0.05, with t test).
